# Generation of a Genome Scale Lentiviral Vector Library for EF1α Promoter-Driven Expression of Human ORFs and Identification of Human Genes Affecting Viral Titer

**DOI:** 10.1371/journal.pone.0051733

**Published:** 2012-12-12

**Authors:** Dubravka Škalamera, Mareike Dahmer, Amy S. Purdon, Benjamin M. Wilson, Max V. Ranall, Antje Blumenthal, Brian Gabrielli, Thomas J. Gonda

**Affiliations:** University of Queensland Diamantina Institute, Princess Alexandra Hospital, Brisbane, Queensland, Australia; St.Louis University, United States of America

## Abstract

The bottleneck in elucidating gene function through high-throughput gain-of-function genome screening is the limited availability of comprehensive libraries for gene overexpression. Lentiviral vectors are the most versatile and widely used vehicles for gene expression in mammalian cells. Lentiviral supernatant libraries for genome screening are commonly generated in the HEK293T cell line, yet very little is known about the effect of introduced sequences on the produced viral titer, which we have shown to be gene dependent. We have generated an arrayed lentiviral vector library for the expression of 17,030 human proteins by using the GATEWAY® cloning system to transfer ORFs from the Mammalian Gene Collection into an EF1alpha promoter-dependent lentiviral expression vector. This promoter was chosen instead of the more potent and widely used CMV promoter, because it is less prone to silencing and provides more stable long term expression. The arrayed lentiviral clones were used to generate viral supernatant by packaging in the HEK293T cell line. The efficiency of transfection and virus production was estimated by measuring the fluorescence of IRES driven GFP, co-expressed with the ORFs. More than 90% of cloned ORFs produced sufficient virus for downstream screening applications. We identified genes which consistently produced very high or very low viral titer. Supernatants from select clones that were either high or low virus producers were tested on a range of cell lines. Some of the low virus producers, including two previously uncharacterized proteins were cytotoxic to HEK293T cells. The library we have constructed presents a powerful resource for high-throughput gain-of-function screening of the human genome and drug-target discovery. Identification of human genes that affect lentivirus production may lead to improved technology for gene expression using lentiviral vectors.

## Introduction

To fully understand functions of the ∼23,000 protein-coding genes in the human genome, the ability to manipulate their expression under different biological conditions and in a variety of cellular backgrounds is essential. This task can be efficiently tackled using arrayed libraries, in which thousands of agents for gene overexpression or knockdown are confined to relatively small volume in microwell plates. Available siRNA and shRNA libraries for gene knockdown and reported loss-of- function genome screens greatly outnumber gain-of- function screens and tools available for high-throughput mammalian gene overexpression [1]. This is in part a reflection of increased difficulty of generating full length constructs for gene overexpression and introducing them into cells, compared to synthesis and introduction of small siRNA molecules into target cells. We [2] and others [3] have recently demonstrated that these difficulties can be overcome by using Gateway technology to transfer genes from available cDNA and open reading frame (ORF) collections [4], [5], [6], [7] into lentiviral expression vectors.

Lentiviral vectors are the most versatile tool for efficient gene delivery into mammalian cells currently available [8], [9], [10]. They are derived from the HIV genome and use a combination of viral and host factors to transport DNA into the cytoplasm, and then nucleus of target cells where it gets inserted into the host genome. Since nuclear membrane disassembly is not required, they can transduce both dividing and non-dividing cells. Insertion into the host genome removes the assay length restriction associated with transient expression of transfected agents. Lentiviral vectors can be pseudotyped using different envelope elements, such as the VSV-G, making them compatible with a wide range of target cell types. Vector elements necessary for optimal virus production and gene expression in target cell line have been extensively studied [11], [12]. The range of available promoters, reporter genes, selection cassettes, and viral packaging systems is continually increasing.

Choosing the promoter to drive target gene expression in gain-of-function screening is particularly important [13]. The cytomegalovirus immediate-early (CMV) promoter has been the promoter of choice in the large scale *in vitro* human gene overexpression studies reported so far [3], [14], [15], [16]. Like other viral promoters such as the simian virus 40 (SV40) promoter, it drives strong constitutive gene expression and is active in a wide range of mammalian cell types. However, transgenes driven by these promoters often become silenced [2], [17], [18], [19], [20]. The timing and level of silencing differs with the cell line and transgene and is difficult to predict. Although not as strong in some cell lines [2], [13], the human translation elongation factor 1α (EF1α) promoter provides stable long-term constitutive expression of most transgenes tested in human cell lines [2], [21], [22], [23]. We therefore used an EF1α promoter-based lentiviral expression vector to generate a library for overexpression screening of the human genome, containing 17,030 ORFs, representing 14,531 unique genes, arrayed in 96-well plates. The vector was designed so that IRES (internal ribosomal entry site)-dependent GFP is co-expressed with the transgene, enabling us to track transgene expressing cells during virus production and target cell transduction. Virus production was carried out in HEK293T cells. Due to ease of culturing and high transfectability [24], the HEK293 cell line and its derivatives have become the preferred choice for optimization of high-throughput transfection-based technology and *in vitro* lentivirus production [25], [26], [27]. Despite the extensive use of this cell line, the effect of introduced target genes on the viral titers produced remains largely undescribed.

We have previously observed [2] that the obtained viral titers are mostly dependent on the expressed ORF sequence, as we identified genes that consistently produced either very high or very low viral titers. For genes of up to 3.5 kb, this effect was independent of their size. Tracking of GFP fluorescence in the virus producing cell line in this study allowed us to explore this effect on a genome scale. Here we describe generation and analysis of the library and transduction rates on a range of cell lines, and the identification of introduced genes which consistently produced high or low viral titers.

## Results

### CMV and EF1α Promoter Comparison in Stable Cell Lines

**Figure 1:**
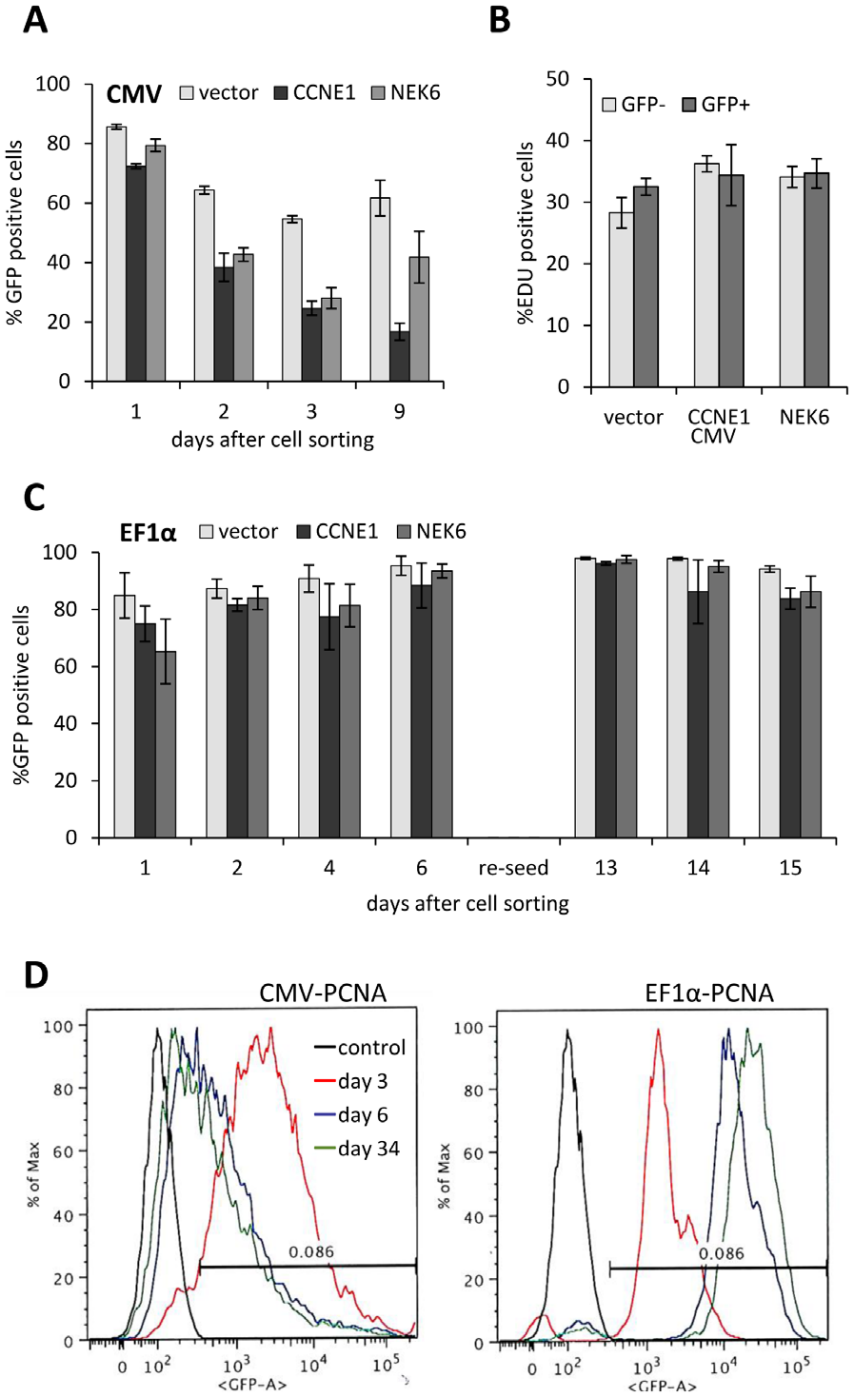
CMV and EF1α promoter comparison in stable cell lines. MCF10A cells expressing human ORF constructs were selected by flow cytometry to minimum 98% GFP positive, seeded and fixed for analysis on indicated day following sorting. A,B,C: Bar graphs representing the proportion of GFP (A,C) or EdU (B) cells obtained by high-content imaging of cells in 96 well plates. A, B: CMV promoter constructs; C: EF1α promoter constructs. All bars represent mean of 4 wells (error bars = SD). D: frequency histograms of cells analysed for GFP fluorescence by flow cytometry, control data (black curves) are for the parental, untransduced cells, black bars indicate threshold used for selecting GFP positive cells during sorting.

Prior to generating a genome-wide overexpression library, we confirmed that the EF1α promoter provides more stable long-term expression compared to the CMV promoter. We treated MCF10A cells with lentiviral supernatant carrying human ORFs driven by either EF1α or CMV promoter. The ORFs were tagged with IRES-GFP so that transduced cells could be tracked by fluorescence. Cells were expanded and sorted by flow cytometry to select GFP positive cells. The selected cells were expanded and sorted again to remove any untransduced cells that passed the first selection round. The proportion of GFP positive cells in the selected population was >98%. Selected cells were seeded into 96-well plates and analysed by high-content imager for several days following sorting. Some cell lines were also analysed by flow cytometry. There was a 10–20% reduction in the proportion of GFP cells in all cell lines within the first 24 h of seeding, possibly due to cell damage during flow cytometry. The proportion of GFP positive cells was reduced by 30–60% within 2 days following sorting in cell lines expressing CMV promoter-driven NEK6, CCNE1 and PCNA (Figure 1A and 1D). This reduction was not due to proliferative disadvantage of transduced cells as both the GFP positive and negative cells had similar EdU incorporation rates (Figure 1B). In contrast, the proportion of GFP positive cells remained high (above 80%) in cell lines expressing the same genes driven by the EF1α promoter for up to 15 days for NEK6 and CCNE1 (Figure 1C) and 34 days after sorting for PCNA (Figure 1D). We have previously demonstrated that the NEK6 and CCNE1 construct-transduced cell lines were expressing increased amounts of introduced protein using Western blots [2].

### Library Construction and Analysis

**Figure 2:**
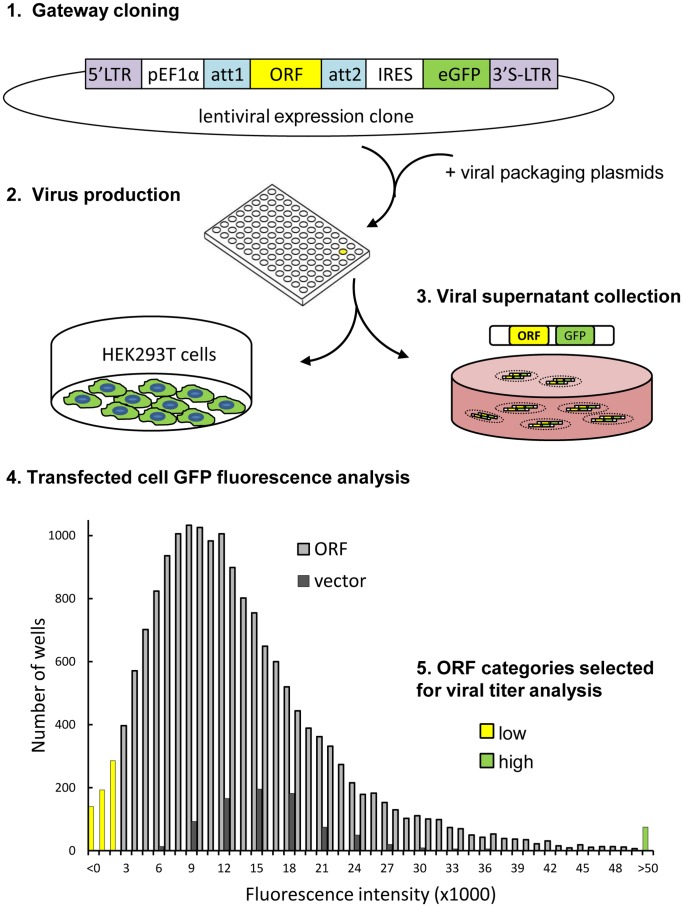
Diagram summarising the key library construction steps. 1. Gateway cloning was used to transfer ORFs into the lentiviral expression plasmid plv411G downstream of the EF1α promoter and upstream of the IRES driven GFP; 2. Virus production was performed by transfecting expression clones together with viral packaging plasmids into HEK293T cell line; 3. Viral supernatant was collected into a fresh set of plates and stored; 4. Transfected cells remaining in the plates were fixed and scanned to determine GFP fluorescence in each well. Frequency distribution histogram illustrates number of wells (y-axis) that had similar GFP fluorescence intensity (x-axis). Values between plates were normalized by zeroing on the mean of 4 mock transfected wells on each plate. Each plate also contained 4 empty expression vector wells, shown in green, which were used as positive controls; 5. Thirty eight ORF-expressing wells were randomly selected from each of the tail ends of the frequency distribution categories, to evaluate the performance of the viral supernatants on a range of target cell lines.

In order to generate a resource for genome-wide, overexpression screening in mammalian cell lines, we acquired arrayed Gateway-compatible entry clones for 17,030 open reading frames (ORFs) from the Mammalian Gene Collection sourced from the hORFeome v5.1 and ImaGenes collections (Table S1) [4], [5], [6]. The library consists of 190, 96-microwell plates which were processed on a liquid-handling robotic platform as summarized in Figure 2 and detailed in Figure S1. ORF’s from the entry clones were inserted into a lentiviral expression vector plv411G [2], [28] between the EF1α promoter and IRES-dependent GFP (Figure 1. Step 1.), so that the ORF is expressed from the same transcript as GFP, while the resulting proteins and translation frames remain independent. As the entry plasmid DNA originated from a cloned PCR product and single clone isolation on this scale was impractical, we modified the Gateway LR-clonase reaction, to increase the likelihood of obtaining a single species of DNA in the resultant expression clone. The amount of entry plasmid DNA in the reaction was reduced to below 5 ng while the destination plasmid DNA was maintained at 100 ng per well. Sample sequencing of single colony isolates from 20 expression clone wells, confirmed that up to 95% of expression clone wells contained uniform DNA species, based on sampling 4–8 colonies per clone (data not shown). Single pass insert sequencing of 275 sample expression clones, resulted in 259 (94%) traces with correct insert identity (Table S2), 6 that matched a gene assigned to a different well, and 7 with low quality sequence due to either overlapping traces, or poor signal intensity (Table S2). Gateway cloning and expression plasmid DNA preparation were performed on the 190 plates. Prior to virus production, the library was reformatted into 205 plates so that one column on each plate could be freed for control wells. For viral production the control column contained 4 empty vector wells (expressing GFP but no ORF), and 4 mock wells which contained no expression plasmid.

**Figure 3:**
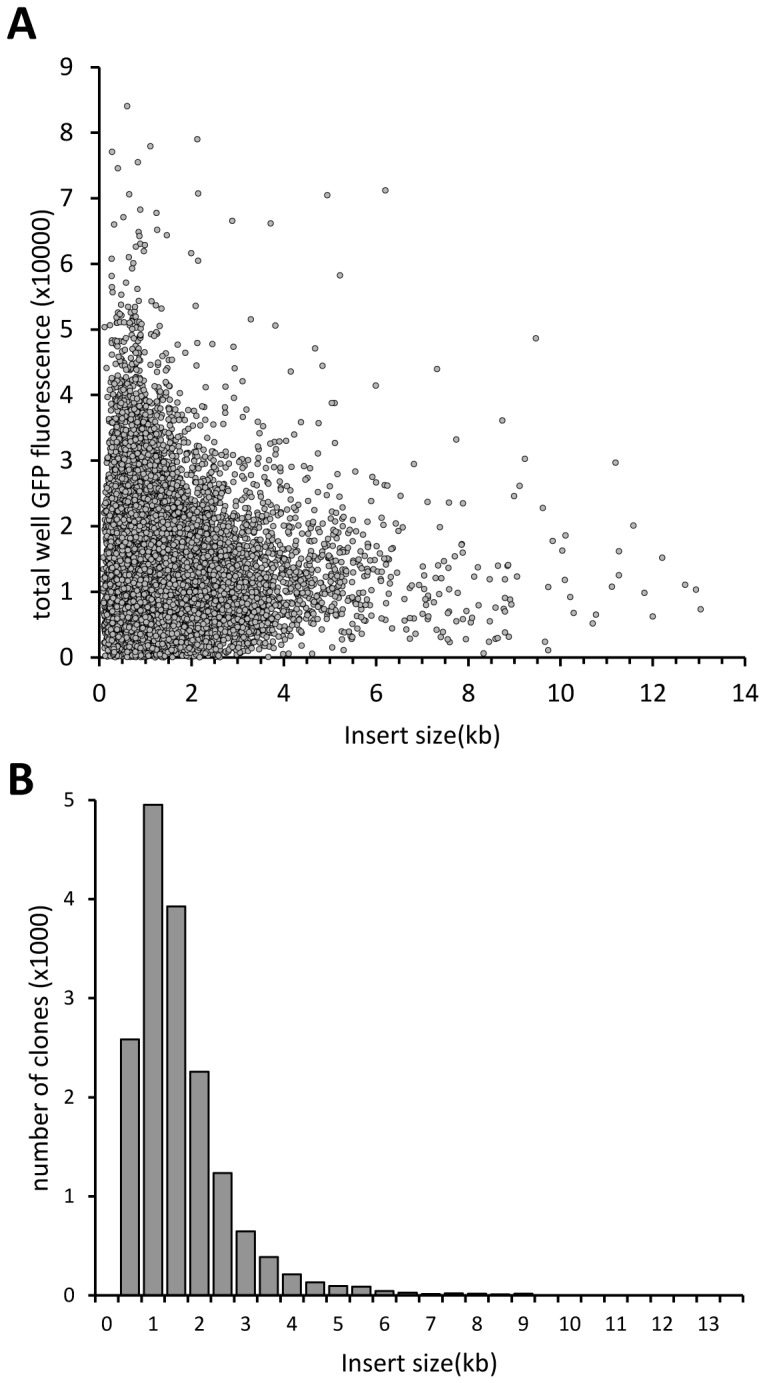
Library clone insert size and GFP fluorescence in virus-making cells. A: scatter-plot of cloned insert size and total well GFP fluorescence in transfected HEK293T cells. B: frequency distribution histogram of clones in different insert size category.

Expression clones were then combined with lentiviral packaging plasmids [29] and transfected into HEK293T cells to produce viral supernatant on a separate high-throughput platform (Figure 2, step 2). HEK293T cells are easily transfected and produce good viral titers, but are poorly adherent and easily dislodged from the plates during processing, potentially contaminating the viral supernatants. To overcome this problem we coated the wells with gelatin before cell seeding. This improved cell retention in the wells and allowed us to assay the transfected cells for GFP expression as an estimate of the viral titer in the collected supernatant. After the removal of viral supernatant, transfected cells were fixed and whole well GFP fluorescence intensity measured using a fluorescence plate reader. As the background fluorescence varied between batches of plates, we used the mean of the fluorescence intensity values from the 4 mock wells to zero the data from each plate and normalize it for plate to plate comparison. The results are summarized in the frequency histogram in Figure 2 (for individual ORF values see Table S1). Fluorescence intensity above 2464 (two standard deviations above the mock), was observed in 16,239 sample wells, indicating that 95% of transfected ORFs produced some GFP positive cells. More than 90% (15,412) of sample wells had fluorescence intensity above 4,058, the lowest value observed for the empty vector. The empty vector routinely produces viral titers of 10^5^–10^6^ pfu per ml, corresponding to 60–90% (depending on cell density and type) target cell transduction rate in downstream screening. Library clones ranged in predicted insert size from 75–13037 bp. For inserts of up to 3500 bp there was no correlation between insert size and total well GFP fluorescence intensity of virus producing cells (Figure 3). This represents the majority of library wells, as only 703 ORFs (4.1%) were outside this size limit (Figure 3B). For practical reasons, the amount of expression plasmid DNA added to each well was normalized by actual mass, not molecular weight, so that there were more copies of the plasmids with smaller inserts in the transfection mix. Given that the vector backbone is 9.134 kb, for the majority of clones the insert contributed a relatively small proportion of the molecular weight of the plasmid. Therefore the effect of insert size on the number of plasmid copies each cell received was greatly attenuated. Although there were no ORFs larger than 10 kb in the highest GFP fluorescence intensity category (above 50,000), they were also under-represented in the library. Therefore, it cannot be concluded that this is a general trend without further experiments.

### Identification of ORFs Producing Very Low or Very High Viral Titer

**Figure 4:**
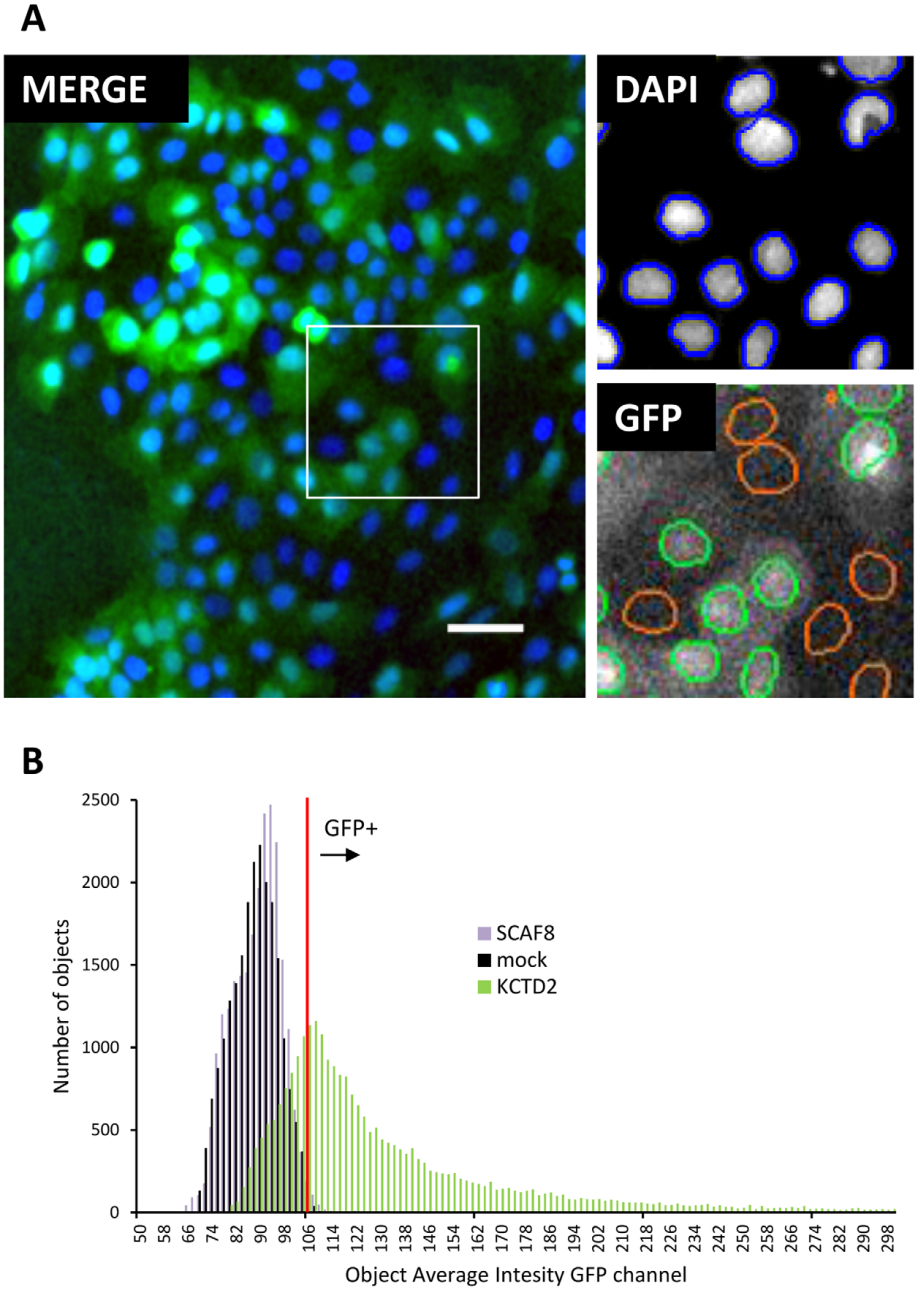
High-content image analysis. A- Pseudo-coloured overlay (MERGE: GFP channel green, DAPI channel, blue, bar  = 50 µm) of images from one scanned field within a well and object analysis masks for the enlarged boxed area in DAPI and GFP channels. DAPI channel was used to determine nuclear area mask (blue outline). Nuclear area was then analysed for average pixel intensity in the GFP channel, and threshold set visually to distinguish GFP positive (green outline) from GFP negative nuclei (orange outline). B-Average nuclear GFP pixel intensity frequency histograms for wells representing untransduced (mock), or cells transduced with low (SCAF8) or high (KCTD2) titer gene supernatant. Mock well histograms were used to validate and/or adjust intensity threshold (red line) for identifying GFP positive cells. Images and data are from HaCat cell line, taken with a 10x objective, image scanning and analysis was performed using Cellomics TargetActivation v3. application algorithm.

**Figure 5:**
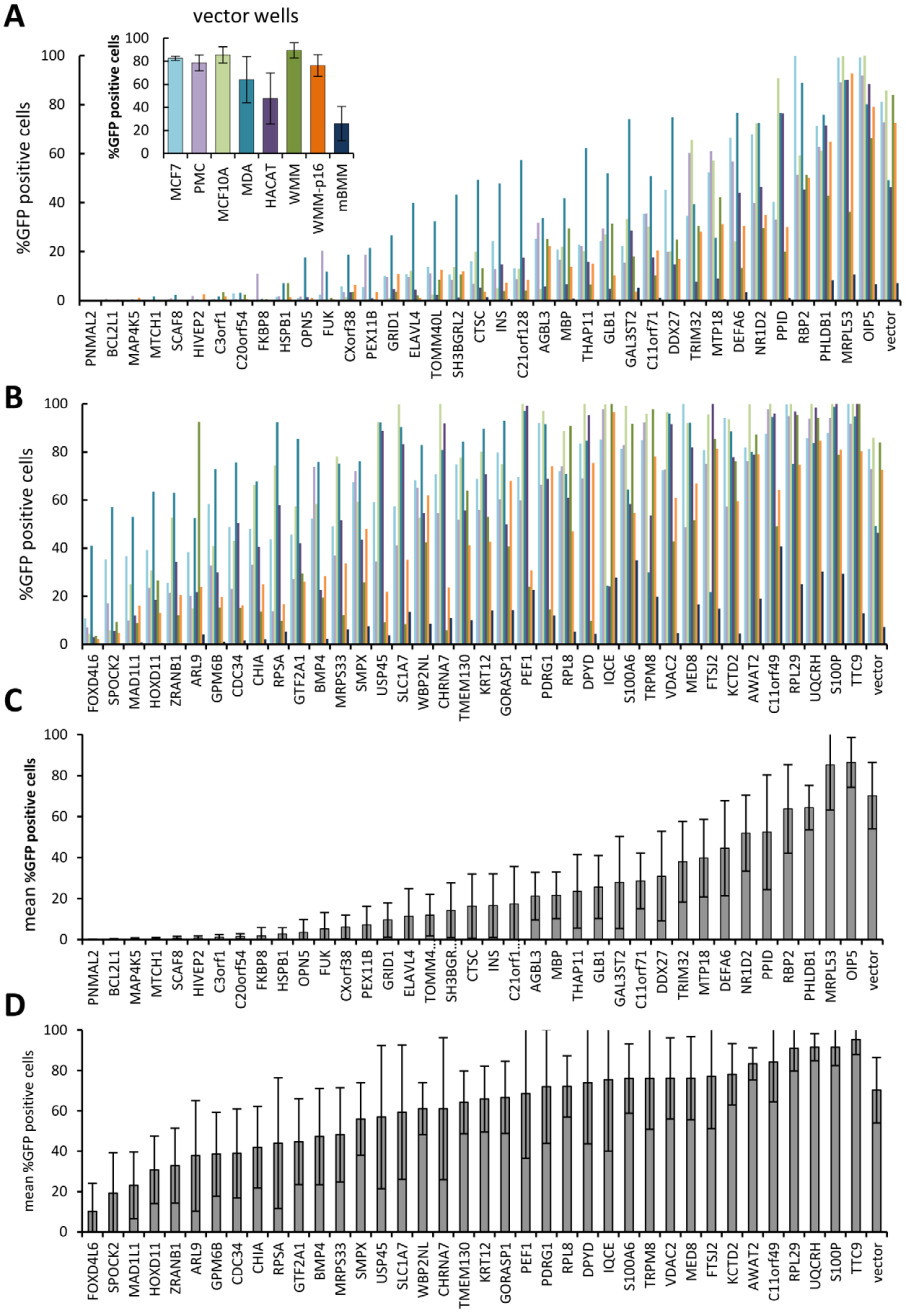
Transduction rates observed with low and high titer virus-producing expression clones. A,C – low titer clones; B,D –high titer clones. The bars in the inset in graph A represent mean and standard deviation (error bars) of four wells transduced with empty vector virus. Color of bars corresponds to the target cell line as indicated in the inset graph. Actively dividing human epithelial tumor (MCF7, PMC42-ET, MDA-MB-468, WMM1175), and non-tumor (MCF10A, HaCaT) cell lines were compared to non-dividing cells (WMM1175-p16, arrested by induced overexpression of p16, and primary mouse bone marrow macrophages (mBMM)). The proportion of GFP positive cells (y-axis) was determined after high-content imaging of plates containing fixed transduced cells. Each bar in A and B represents data for a well transduced with a single gene-expressing vector as indicated on x-axis. Bars representing mean values for vector wells are included in all three graphs to allow for scale comparison. Each bar in C and D represents a mean (error bar  = SD) transduction rate for all human cell lines for a given gene.

To investigate the range of titers produced in our library, and its consequences on screening applications, we generated one 96-well plate containing 38 genes which produced highest GFP fluorescence intensity in virus producing cells (above 50,000), and 39 genes selected from the lowest intensity producing wells (below 3,000). For the low category, we excluded wells that did not contain expression clone DNA in the detectable range (above 20 ng/µl) (Table S1). Identity of all selected genes was confirmed by sequencing. We produced viral supernatant and tested it on a range of cell lines, scheduled for overexpression screening. We included human mammary (MCF7, MCF10A, PMC42ET, MDA-MB-468) and skin (HaCaT, WMM1175) epithelial actively dividing cell lines. Except for MCF10As and HaCaTs, the other human cell lines were derived from tumors and are aneuploid and tumorigenic. We also tested the ability of the virus to transduce non-dividing cells by infecting WMM1175 expressing inducible p16^Ink4a^ [30] which stably arrests cells in G1 phase when induced, and mouse primary bone marrow macrophages which were not proliferating under conditions used and which also served as a cross-species infectivity test. Cells were transduced in 96-well plates, incubated, and fixed. After staining with DAPI, to visualize cell nuclei, plates were scanned using a high-content imager. Number of nuclei was determined in the DAPI channel and GFP status for each nucleus determined in the GFP channel as illustrated in Figure 4. GFP fluorescence intensity histograms for untransduced wells were used to confirm validity of GFP thresholds set by visual inspection of the images. As before, 4 empty vector wells on each plate were used for control. The results are summarized in Figure 5, and complete data are presented in Table S3.

Transduction of a range of human cell lines with empty vector virus resulted in GFP expression in a high proportion of cells in all cell lines tested (Figure 5A, inset). Transduction rates in HaCaT and MDA-MB-468 cell line were lower, averaging 47% and 64%, compared to other cell lines which averaged around 80%. This indicates that although there may be some variability between cell lines, the lentiviral library is suitable for screening a broad range of cell lines with little modification of the transduction protocol. There was no significant difference in observed transduction rates between actively dividing and arrested WMM1175 cells, confirming the ability of lentiviruses to infect non-dividing cells. The proportion of GFP positive cells observed in the transduced mouse primary bone marrow macrophages was too low to make screening in these cells practical. However, since 32 of the high titer and 7 of the low titer-producing ORFs produced over 2% GFP positive cells, the library supernatants could be used in downstream (hit-validation) experiments in a mouse model if necessary.

Despite a considerable difference in transduction rates between genes and in some cases between different cell lines transduced with the same gene, some reproducible trends could be detected. Supernatant from transfection of genes that induced the lowest GFP fluorescence in packaging cells (“low titer genes”) (Figure 5A, 5C), generally resulted in lower transduction rates compared to the supernatant from the highest GFP producers (“high titer genes”) (Figure 5B, 5D). Among the low titer genes, 9 genes (HIVEP2, MAP4K5, BCL2L1, MTCH1, C3ORF1, PNMAL2, RBM16 (SCAF8), C20ORF54) produced very low numbers of GFP positive cells in all cell lines tested, while 6 genes (OPIP5, MRPL53, PPID, NR1D2, TRIM32, PHLDB1) produced transduction rates comparable to those observed with high titer genes, achieving nearly 100% GFP positive cells in some cell lines. This observation suggests that not all of the HEK293T wells in which GFP could not be detected, failed to produce virus. Except for FOXD4L, all of the high titer genes produced a high proportion (above 50%) of GFP positive cells in at least one of the human cell lines tested, and 33 of the 39 reached rates of 80% or more, suggesting that high level of GFP fluorescence in virus-producing cells is a good indicator of high viral titer in the supernatant. Cell numbers in wells treated with supernatants from all genes were reduced compared to the wells treated with mock supernatant. Although the degree of this effect varied between genes, there was no significant difference between low and high titer genes as a group (Table S3). We have previously demonstrated that this effect is at least in part due to the reduced proliferation in transduced cells [2].

As observed previously [2], there was no correlation between insert size and virus titers produced although ORFs larger than 3.5 kb generally had lower virus production. Genes in the high titer group ranged from 0.2 to 2.2 kb. With the exception of HIVEP2 (7.4 kb) and RBM16 (SCAF8, 3.8 kb), genes in the low titer group ranged from 0.3 to 2.9 kb. We performed functional clustering using the DAVID functional annotation web interface [31], to determine if there were any functional categories enriched in our high and low titer producing genes. As the background gene list we used either our ORF library or the whole human genome. Both analyses provided similar results except that enrichment levels were slightly lower and P values slightly higher when the ORF library list was used as background. The values with the ORF library as background list are cited here. In the low titer producing genes, groups enriched at P<0.05 were genes encoding mitochondrial proteins (MRPL53, PEX11B, FKBP8, C3ORF1, TOMM40L, MTCH1, BCL2L1, MTP18; 4.7 fold enriched at P = 0.001) and cell death genes (FKBP8, INS, MTCH1, HSPB1, BCL2L1, MTP18; 4.1 fold enriched at P = 0.01). In the high titer genes, groups enriched were ribosomal subunit genes (RPSA, MRPS33, RPL8, RPL29; 11.5 fold, at P = 0.004), protein dimerization activity (BMP4, PEF1, S100A6, TRPM8, GTF2A1, CHRNA7, DPYD; 5.1 fold at P = 0.001), EF-hand type, calcium binding (PEF1, S100A6, S100P, SPOCK2; 7.8 fold, at P = 0.001). At the time of writing, DAVID tool could not assign function to three of the low titer genes (CXORF38, C21ORF128, C11ORF71) and one high titer gene (C11ORF49). GFP fluorescence intensity histograms showed that GFP intensity varied by several orders of magnitude between cells within a well transduced with a single high titer gene (eg. KCTD2, Figure 4B). This is possibly due in part to varying multiplicities of infection (MOI) between cells, resulting in multiple proviral integrations in some cells so that the actual titer for these genes may be even higher than suggested by the observed transduction rates.

**Figure 6:**
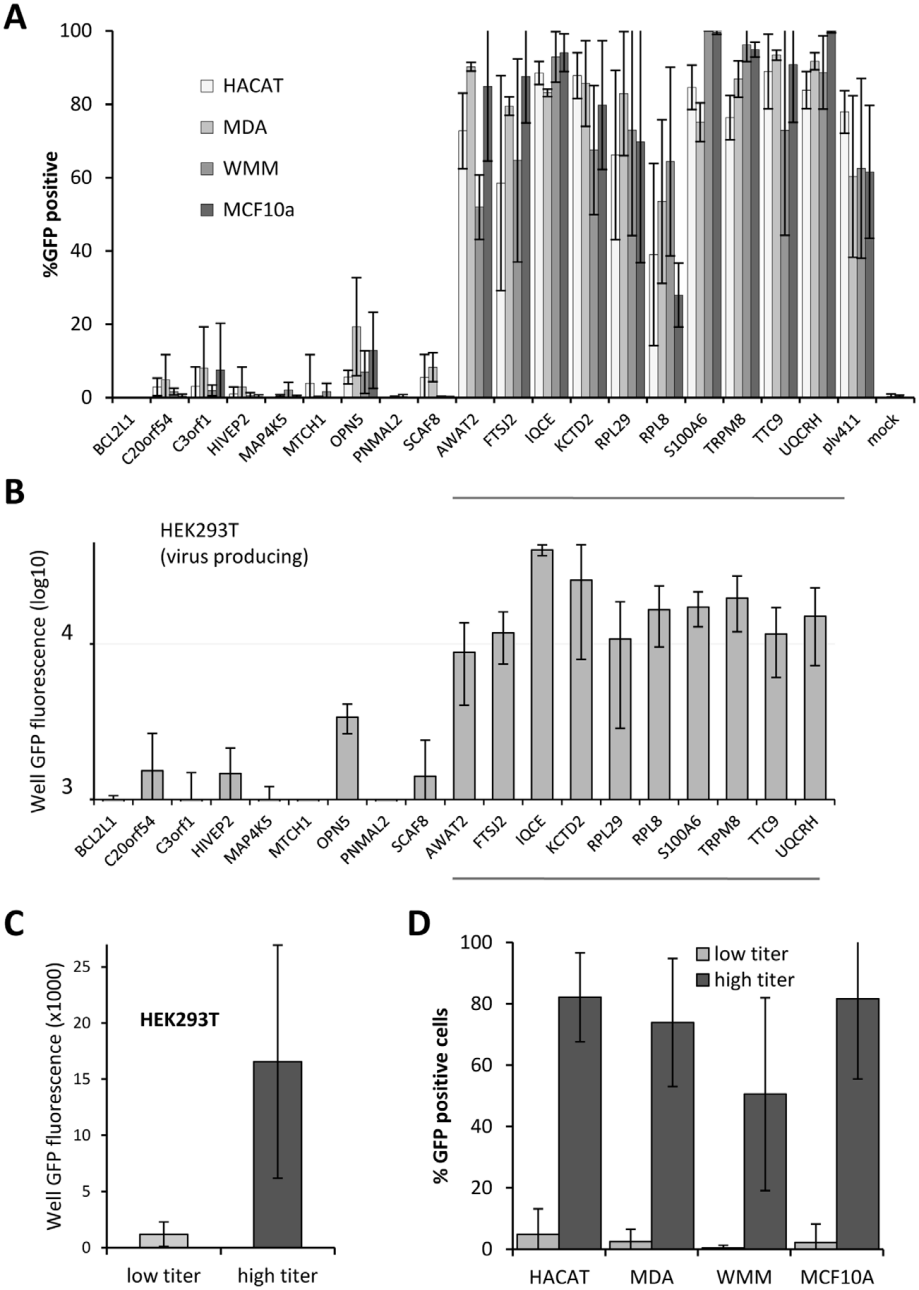
Comparison of target cell transduction rate and GFP fluorescence of corresponding virus-producing wells. A,C – target cell transduction rates; B,D – GFP fluorescence of virus producing wells. Bars in A and B represent mean of 4 wells derived from independent single colony isolates of the gene-expressing plasmids (x-axis). Genes underlined in A, produced high viral titer in bulk well experiments (Figures 1 and 2.), the rest were low titer producers. Error bars = SD. Data for graphs A and C were obtained by high-content imaging of transduced cells, while data for graphs B and D were obtained by scanning the transfected cells using a fluorescence plate reader. C and D: Mean and SD for high and low titer well data represented in A and B. In all cell lines mean for combined low titer genes was significantly different from the mean for high titer wells (Aspin-Welch test, P value: HEK293T = 4.70 E−11; HACAT = 5.68 E−23; MDA-MB468 = 2.32 E−36; WMM1175 = 7.29 E−12; MCF10A  = 2.98 E−21).

To investigate the reproducibility of the viral titers produced by individual genes, we selected 19 genes (9 with the lowest titer, 10 with the highest titer, Table 1), and prepared expression clone DNA from four single colony isolates for each gene. Inserts for each clone were fully sequenced and compared. All four clones were 100% identical in nucleotide sequence for 17 of the 19 investigated genes. For C3ORF1 one of the 4 clones contained a 2 nucleotide deletion close to the 3′ terminal (nucleotides AA at positions 841 and 842 of 857, accession BC013999). The clone containing the deletion did not behave differently to the others in subsequent analysis. For IQCE, 2 of the 4 isolated clones, contained an ampicillin resistant plasmid that failed to sequence with primers targeting ORF insertion site. They did not produce any GFP positive cells, suggesting that they were either a cloning artifact or a contaminant. They were excluded from further analysis. Viral supernatant was generated from the re-isolated expression clone DNA and used to transduce four target cell lines, as above. Results are presented in Figure 6.

**Table 1:** Genes producing very high or very low viral titer.

Titer	Symbol (synonyms)	Description	size (bp)	ACCESSIONS (parent clone)
low	BCL2L1 (BCL-XL)	BCL2-like, apoptotic regulator	702	BC019307
	C20orf54 (SLC52A3,RFT2)	solute carrier family 52, riboflavin transporter, member 3	1410	BC009750
	C3orf1 (TIMMDC)	translocase of inner mitochondrial membrane domain containing 1	858	BC012341
	HIVEP2 (SHN2,ZAS2)	transcription factor, HIV type 1, enhancer binding protein 2	7402	NM_006734.3
	MAP4K5 (KHS, GCKR)	mitogen-activated protein kinase kinase kinase 5	2541	BC036013
	MTCH1(PSAP, PIG60)	mitochondrial carrier 1	927	BC000702
	OPN5 (PGR12, GPR136, TMEM13)	opsin 5, G-protein coupled receptor	1098	BC126194
	PNMAL2 (FLJ42730, KIAA1183)	paraneoplastic MA antigen family-like 2	612	BC132839
	SCAF8 (RBM16)	SR-related CTD-associated factor 8, RNA binding protein 16 mRNA processing, RNA splicing	4047	BC070071
high	AWAT2 (WS, DC4, MFAT)	acyl-CoA wax alcohol acyltransferase 2	1062	NM_001002254
	FTSJ2 (FJH1)	FTSJ homolg 2	741	BC121109
	IQCE	IQ motif containing E	251	BC071858
	KCTD2 (KIAA0176)	potassium channel tetramerisation domain containing 2	853	NM_015353.1
	RPL29 (HIP, L29, HUMRPL29)	ribosomal protein L29, also cell surface heparin binding	522	BC008926
	RPL8 (L8)	ribosomal protein L8	774	BC000077
	S100A6 (2A9, PRA, 5B10, CABP, CACY)	S100 calcium binding protein A6	272	BC001431
	TRPM8 (TRPP8, LTRPC6)	transient receptor potential cation channel, subfamily M, member 8	578	BC001135
	TTC9 (TTC9A)	tetratricopeptide repeat domain 9	900	NM_015351.1
	UQCRH (QCR6, UQCR8)	ubiquinol-cytochrome c reductase hinge protein	279	BC001426

Transduction rates (Figure 6A) were obtained by determining the proportion of GFP positive cells in the virus treated cells using a high-content imager. Except for OPN5 transduction of MDA-MB-468 cells, all low titer genes had transduction rates of less than 20% in all four cell lines tested. Most high titer genes had transduction rates above 60% in all cell-lines tested, except for AWAT2 on WMM1175 (52%) and RPL8 on MDA-MB-468 (51%) and MCF10A cells (29%). Similar patterns were observed in total GFP fluorescence of HEK293T cells used to produce the virus (Figure 6B), where values of less than 5,000 were observed for all low titer genes except for OPN5 which was 8,800. In contrast, means for total well fluorescence for high titer genes ranged between 12,420 for empty vector plv411 and 43,087 for S100A6 (Figure 6B). The considerable variability between individual wells for each gene in the high titer group is due to dislodging of virus producing cells during plate processing and uneven distribution of remaining cells. Statistical analysis of the combined means for all low and high titer gene revealed that they were significantly different in both proportion of GFP positive cells in transduced cell lines (Figure 6C) and total well GFP fluorescence in transfected virus producing cells (Figure 6D). Using Aspin-Welch test, P values for the difference between means between low and high titer genes were: HEK293T,  = 4.70 E−11; HACAT  = 5.68 E−23; MDA-MB468 = 2.32 E−36; WMM1175 = 7.29 E−12; MCF10A  = 2.98 E−21. Together these data suggest that achieved viral titers are gene-specific and reproducible. There was no general difference between the low and high titer genes in the total number of cells remaining in the well 3 days after transduction in all four transduced cell lines tested (data not shown), indicating that the observed reduction of GFP positive cells in the cell lines transduced with the low titer genes is not due to the their deleterious effect on transduced cells.

**Figure 7:**
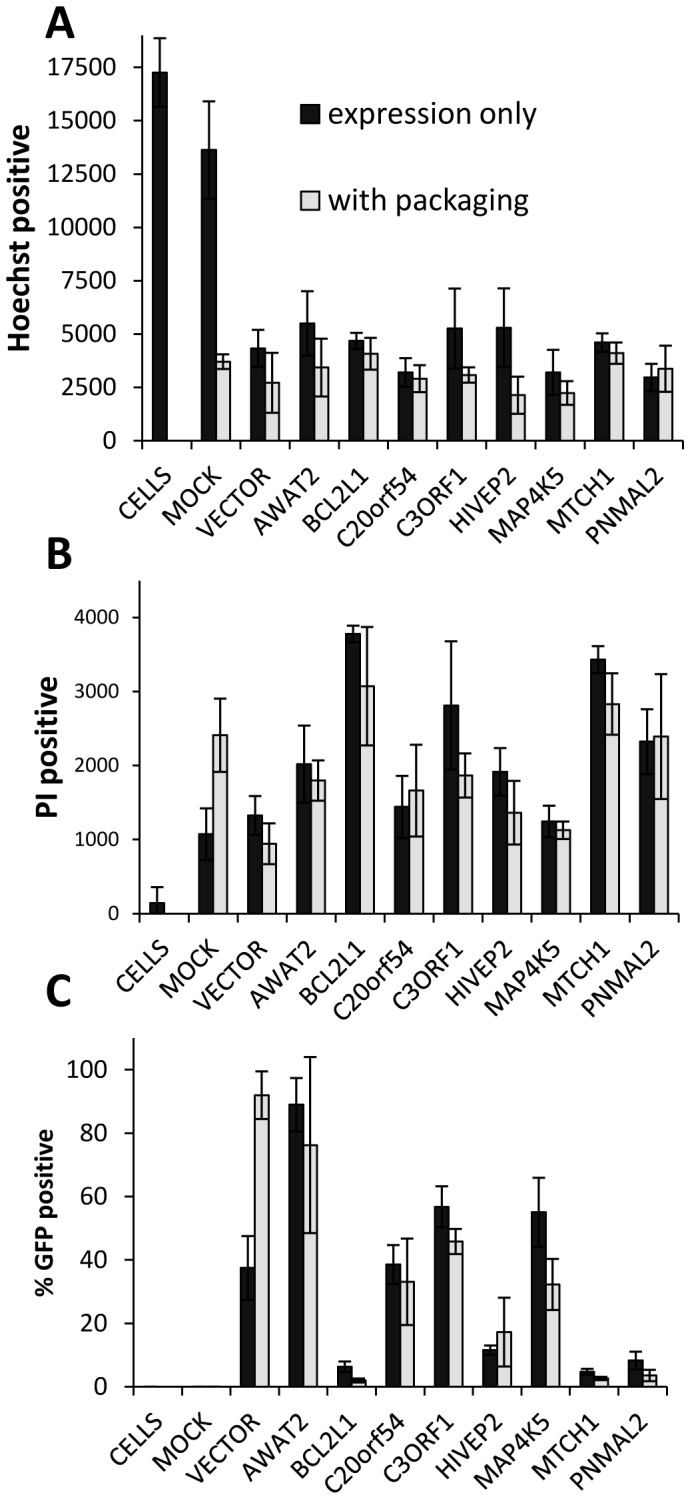
Effect of low virus titer-producing genes on viability of the transfected cells. HEK293T cells were transfected with gene (x-axis) expressing plasmids either alone or with viral packaging vector day after seeding. 21 h after transfection, medium was replaced with PBS containing Hoechst33342 and propidium iodide (PI), which stain DNA and dying cells respectively. Cells were scanned in blue (Hoechst, A) red (PI, B) and green (GFP, C) channels and number of objects determined in each channel. A and B represent independent object counts, data in C are expressed as proportion of GFP positive cells in the Hoechst stained population. A - Significantly reduced number of surviving cells in the well compared to untreated wells (CELLS): lipofectamine alone (MOCK) (P = 0.034); MOCK with packaging plasmids (P = 7.5E−10); all others (P≤1.1E−05) B - Significantly increased PI positive cells compared to vector: expression plasmid only BCL2L1 (P = 6.88E−05), C3ORF1(P = 0.03), MTCH1(P = 4.66E−05), PNMAL2 (P = 0.01) P values determined using Aspin-Welch test; Bars = mean of 4 wells, error bars = SD, CELLS-untreated well, MOCK-wells where expression plasmid has been omitted. AWAT2, and vector were high virus titer producing in previous experiments.

Since the functional annotation suggested that cell death genes were enriched among the low titer genes, we investigated the effect of these genes on virus producing HEK293T cells. We used propidium iodide (PI) staining to determine the number of dead cells following transfection. To separate the effect of viral particle production and release from the effect of gene expression, we transfected cells with ORF expression plasmids with or without addition of viral packaging plasmids. Live cells were imaged in three channels (blue for nuclear stain, red for dead cells, and green for GFP positive cells), 21 h after transfection. The results are presented in Figure 7. Transfection with packaging vectors alone, significantly reduced the number of surviving cells as indicated by the reduced number of Hoechst positive nuclei (Figure 7A), and increased number of cells losing membrane integrity as indicated by PI staining (Figure 7B). In addition cells transfected with expression plasmids carrying BCL2L1, C3ORF1, MTCH1 or PNMAL2 without packaging plasmids had significantly increased number of PI positive cells, compared to cells transfected with the empty vector (P value: BCL2L1 = 6.88E-05, C3ORF1 = 0.03, MTCH1 = 4.66E−05, PNMAL2 = 0.01), or other genes, including AWAT2, which was a high-titer producing gene.

## Discussion

We have generated an important new resource for gain-of-function screening of the human genome. In comparison with previously reported human gene lentiviral expression libraries it contains more clones and as far as we are aware, it is the only public large scale library not driven by the CMV promoter. The use of the constitutive human EF1α promoter in our library increases the range of cell types and the length of assays that can be used in screening as we have demonstrated that the EF1α promoter provides more stable long term expression in the MCF10A cell line. Since the CMV promoter silencing is a well-documented phenomenon [17], [18], [19], [20] we have not investigated the mechanism behind this observation. The EF1α promoter has been shown to be superior driver of long term transgene expression compared to the CMV promoter in a number of other mammalian cell types [13], . Although our library is not derived from single clone isolates of the introduced ORFs, sample sequencing indicated that 95% of the wells contained a single insert of correct sequence. This suggests that our strategy of diluting entry clone DNA used in the Gateway LR reaction to 1 ngµl^−1^ or less, was generally successful in selecting a single DNA species from the PCR derived inserts in the entry clones.

Analysis of GFP fluorescence in virus-producing HEK293T cells indicated that at least 90% of ORF clones were successfully transfected. The level of detected GFP fluorescence varied between wells but the intensity level was generally not related to inserted ORF size. Although not strictly correlated, GFP fluorescence of virus producing cells was a good general indicator of viral titers in the collected supernatant, as demonstrated by assessing transduction rates of selected clones on a range of cell lines. We used both tumorigenic and non-tumorigenic human cell lines and tested one non-dividing human and one mouse cell line. Most of the genes which produced high GFP fluorescence in virus-producing cells, also gave high transduction rates, while only about two thirds of the low GFP fluorescence-producing genes also gave low transduction rates. This high rate of false negative results is most likely due to the fact that virus making cells are easily dislodged during supernatant collection, so that some wells where transfection rates were high gave a blank reading due to cell loss. Although not detected in our study, a small number of false positive results could arise when using GFP expression in transfected cells as an indicator of viral particle production due to the reduced packaging efficiency that has been observed for lentiviral expression vectors with increased proviral length [33]. Since this would substantially affect less than 4% of clones in our library, we have not investigated it further. The observed transduction rates on the nine human cell lines suggest that our library of supernatants can be used for screening a wide range of cell lines and that functional data can be generated for up to 15,412 human ORFs, since they had GFP fluorescence above that of the minimum observed for the empty vector plasmid which routinely resulted in transduction rates of above 60%. The library supernatants have so far been used in two genome wide screens: on the melanoma WMM1175 cells arrested by induced expression of p16^Ink4a^ protein and on the breast cancer MDA-MB-468 cells carrying a reporter construct. In these screens respectively 85 and 93% of valid transduced wells yielded more than 50 GFP positive cells (unpublished observations). Transduction rates obtained on the primary mouse bone marrow macrophages were insufficient to make screening practical in this cell line. The observed transduction rates of some of the genes were sufficient for low throughput assays indicating that function of at least some of the genes can be assessed in mouse macrophages using our lentiviral clones. This may prove as a useful alternative for gene delivery into these cells, given that primary mouse macrophages are notoriously difficult to transfect [34], [35].

We have identified genes that consistently produced either high or low lentiviral titers in multiple rounds of supernatant generation. We determined viral titer in terms of the proportion of transduced cells obtained by infecting with the collected supernatant at a single dilution to allow for the analysis of large number of genes. The total amount of GFP fluorescence of transduced cells has been shown to be directly correlated to the amount of lentiviral vector RNA in the viral supernatant in a study evaluating lentiviral vector titration methods [36]. Viral titers for the high titer genes in our study are likely to be underestimated because we have not assayed diluted supernatants or measured MOI. GFP fluorescence of the transduced cells can also be used as indicator of the ORF gene expression since GFP is positioned after the IRES on the same transcript, and protein levels of the second gene in this type of bicistronic vector are generally lower than that of the first gene [37]. We have previously confirmed transgenic ORF expression of select clones using Western blots and/or immunocytochemistry [2]. Since most of the low and high titer producer genes were smaller than 3 kb, the achieved titer appeared to be a consequence of specific gene sequence, rather than ORF size. It has long been known that lentiviral particle production and subsequent infectivity depends on both viral and host cell factors that could be acting at either RNA or protein level. In our study ORF transcript level and stability in virus producing cells is likely to be enhanced by cis-acting elements in the vector backbone such as the central polypurine tract and the hepatitis B posttranscriptional regulatory element [28], [38]. These factors, together with promoter activity are likely to affect all ORF transcripts equally. Although it is still possible that the differences in viral titer we have observed are due to differences in RNA stability or packaging efficiency, our data suggest that overall it is more likely that they are due to the protein function. Several studies have identified a range of human proteins that can affect the life cycle of the HIV lentivirus [39], [40], [41], either through specific interaction with viral proteins, or through general effects on gene transcription, protein synthesis and cell metabolism. Although the lentiviral vectors originated from this virus, very little is known about the host factors affecting lentiviral vector production and how they relate to native viral infection since many of the native viral sequences have been removed from the expression and packaging vectors. Except for BCL2L1 [40] and HIVEP2 [42], [43] none of the genes identified in our study have so far been implicated in host cell-virus interaction. Even these two genes may have different mechanisms of action during lentiviral vector production compared to HIV infection. During HIV infection BCL2L1 is regulated by viral proteins which are not present in our vector system (env, nef, vpr and tat [40]). Although it is possible that HIVEP2 binds to the enhancer elements in the LTR in our vector as in the native HIV, it is also possible that it failed to produce virus due to its large size (7.4 kb).

We have identified at least four genes which failed to produce virus by increasing death rates in the virus-producing cells. Among them the large isoform of BCL2L1 (BCLX_L_) [44]and MTCH1 (PSAP) [45] have previously been implicated in apoptotic cell death and PNMAL2 shares sequence homology with PNMA proteins implicated in neuronal cell death [46], [47]. It should be noted that BCL2L1 large isoform can have both anti and pro-apoptotic function depending on level of expression and post-translation modification [44], [48], [49]. We were unable to find reports implicating C3ORF the fourth cytotoxic gene in our study, in cell death. Based on the present study, it is impossible to conclude if C3ORF is also a death gene or becomes detrimental only when overexpressed. Overexpression-induced gene cytotoxicity has been observed in all eukaryotic cell types, but has been systematically studied only in yeast, *Drosophila* and *C. elegans* [50]. In these organisms, about 85% of genes which become toxic when overexpressed are normally constitutively expressed, indicating that the cytotoxicity was induced by high gene dosage rather than intrinsic toxicity [51]. Irrespective of the mechanism by which the overexpressed ORFs killed the virus-producing cells, they should be noted as they would give false negative results during functional genome screening. This is especially true of genes cytotoxic to HEK293Tcells, given their wide use in high-throughput screening.

Genes that produced high viral titer included genes that affect lipid synthesis (AWAT2 [52]), protein synthesis (RPL8, RPL29 [53]), mitochondrial function (IQCE, UQCRH [54]), nuclear transport (S100A6 [55]), RNA processing (FTSJ2 [56]) and ion transport (TRPM8 [57], KCTD2), although only the genes involved in protein synthesis and protein dimerisation were enriched as a functional category group. All of these processes have been implicated [10] in lentiviral particle production, assembly and release from the host cell, and it is possible that the overexpression of the high viral titer genes stimulates these cellular functions. Future experiments involving virus production by cells stably expressing these genes will determine which of these genes alter cellular processes so that virus production is increased for all subsequently introduced sequences. Our findings suggest that it will be possible to genetically modify virus producing cell line to improve lentiviral vector yield.

### Conclusions

The 17,030 clone lentiviral vector library for EF1alpha promoter- driven expression of human ORFs represents a valuable new resource for functional identification and analysis of human genes. Identification of genes that strongly effect virus production in the HEK293T cells suggests a potential new approach for further improvement of the lentiviral expression vector technology.

## Materials and Methods

### Ethics Statement

All mouse procedures performed in this study were performed in accordance with institutional regulations after protocol review and approval by the University of Queensland Animal Ethics Committee (DI/567/09).

Unless otherwise noted, all regents were obtained from Sigma-Aldrich (Sydney, Australia).

### Lentiviral Expression Clone Library Construction

Entry clones in pDONOR223 or pENTR201 vectors were obtained as bacterial glycerol stocks. The main, 15,191-clone Human ORFeome collection version 5.1 (Open Biosystems) was supplemented with 1842 non-overlapping clones from ImaGenes ORFeome set (Source Bioscience, Lifesciences) as listed in Table S1. Entry clones were transferred into plv101g (CMV promoter) or plv411g (EF1α promoter) lentiviral expression vectors [2], [28] (both gifts from Dr Simon Barry, University of Adelaide) using the Gateway LR reaction and ScicloneALH3000 robotic liquid handling platform (Caliper Life Sciences; Hopkinton, MA, USA), as described previously [2].

### Promoter Assessment in Stable Cell Lines

MCF10A cells were seeded in 6-well plates at 60,000 cells per well, and treated with 990 µl of viral supernatant containing 12 µgml^−1^ polybrene, the next day. After 2 days cells were passaged and expanded until cell numbers were sufficient for sorting, usually around 7 days. Cells were sorted and GFP positive cells collected using the MoFlo™ automated cell sorter (DakoCytometry). Cells were expanded and sorted again. GFP positive cells (minimum 98% pure) collected after the second round of sorting were seeded directly into 96-well Viewplates (PerkinElmer) at 2,000 cells per well. Proportion of collected cells was expanded. Plates were prepared for high-content imaging as described previously [2]. Briefly cells were pulse-labeled with 10 µM EdU (Berry and Associates; Dexter, MI, USA) for 2 h, fixed in 3.7% formaldehyde in PBS, and stained with Cy5-azide to detect EdU and 400 nM DAPI to localize nuclei. Plates were scanned using the Cellomics ArrayScan VTI (Thermo Scientific) high-content imager, using a 10x objective and an XF053 filter set. Images were collected and analyzed using TargetActivation.v3. application as described previously [2]. For longer term assessment cells were analysed by flow cytometry using a FACSCanto flow cytometer (Becton Dickinson). Cells were grown in flasks, trypsinized, harvested and fixed in 1% formaldehyde in PBS. Percentage of GFP positive cells was determined by counting cells with fluorescence intensity above threshold determined by scanning untransduced cells.

### Cells and Culture Conditions

HEK293T (Broad Institute, Cambridge MA) cells were maintained under standard tissue culture conditions in DMEM supplemented with 10% (v/v) heat-inactivated fetal calf serum (FCS) (Hyclone), 0.85 mM HEPES, 2 mM L-glutamate, 1 mM sodium pyruvate, 1X non-essential amino acids (GIBCO).

MCF-10A cells (ATCC) were maintained in DMEM/F12 (1∶1; Invitrogen) supplemented with 5% (v/v) heat-inactivated horse serum (Invitrogen), 10 µg/ml insulin, 20 ng/ml EGF, 0.5 µg/ml hydrocortisone (Bayer), 100 ng/ml Cholera toxin, and penicillin/streptomycin 100 U/100 µg/ml (pen/strep) (Invitrogen).

MCF7, MDA-MB-468 and PMC42-LA (St. Vincent’s Institute, Melbourne, Australia) cell lines contained dsRed fluorescent protein gene under the control of Vimentin promoter. MCF7 and PMC42-LA cells were grown in RPMI, 10%FCS, penicillin and streptomycin (pen/strep), while MDA-MB-468 and HaCaT (ATCC) were grown in DMEM, 10%FCS, 2 mM L-glutamate, pen/strep.

WMM1175 [58] contained LacZ-promoter-p16 construct, which was induced by addition of 4 mM IPTG to growth media (DMEM, 10% FCS, 4 mM L-glutamate, 1 mM sodium-pyruvate, 10 mM HEPES, 100 U/100 µg/ml pen/strep).

Mouse primary bone marrow macrophages (mBMM) were differentiated from bone marrow cells from 10 week-old C57BL/6 mice for 7d in DMEM with 20% L-cell conditioned medium, 10% FCS, 2 mM L-glutamine, 1 mM sodium-pyruvate, 10 mM HEPES as previously described [59] and seeded in a 96 well plate in the same medium except with L-cell medium reduced to 10%.

### Virus Production in HEK293T Cells

HEK293T cells were plated in 96-well SpectraPlates TC (Perkin Elmer) coated with 0.1% gelatin, at 50,000 cells in 150 µl per well, using Matrix WellMate (Thermo Scientific). The next day, expression clone DNA (300 ng) was mixed with packaging plasmids pRSV-Rev (56 ng), pMDLg/pRRE (130 ng) and pMD.G (90 ng) [29] in 19 µl per well in Costar 3896 plates. LipofectamineTM 2000 transfection reagent (Invitrogen) was incubated with OPTIMEM (Gibco) (1∶31, v/v) for 20–40 min at RT, prior to adding to the DNA mix at 31 µl per well. 130 µl medium was aspirated from each cell-containing well and 50 µl of DNA-Lipofectamine mix was added. Cells were incubated for 1.5–2 h prior to addition of 130 µl of medium containing 3 mM sodium butyrate. After 48 h, viral supernatant (190 µl from each well) was harvested into Costar 3896 plates and stored at −70°C until use. Cells remaining in transfection plates were fixed with 3.7% formaldehyde in PBS. To measure total well GFP fluorescence (excitation wavelength 488 nm, emission 508 nm), plates were scanned using the SynergyMX micro-plate reader(Biotek Instruments). All liquid handling steps were performed on tissue-culture dedicated ScicloneALH3000 unit.

### Transduction Rate Assays

Cells were seeded in clear-bottom 96-well Viewplates (PerkinElmer), at 2,000 cells/well using WellMate. Next day, medium was aspirated, and 30 µl of viral supernatant containing 11.5 µg/ml polybrene was dispensed to each well using the robot. 1.5–3 h later, 150 µl medium was added. Plates were incubated for 3 days, medium was aspirated and cells were fixed in 3.7% formaldehyde for 10 min, washed in PBS, permeabilized in 0.1% tritonX-100, and stained with 400 nM DAPI in PBS for at least 60 min. Intermediate PBS washes between steps were performed using ELx405 plate washer (BioTek Instruments, Winooski VT, USA). For non-dividing cell assays, WMM1175 cells were seeded in medium containing IPTG, and transduced as above 3 days later, to allow for p16 protein to accumulate and induce cell arrest. The arrest was confirmed by lack of staining with ant-Ki67 antibody (data not shown). Primary mouse cells (mBMM) were treated as above except that they were seeded at 20,000 per well and fixed 8 days after transduction. Images were acquired with a Cellomics ArrayScan VTI (Thermo Scientific) high-content imager, using a 10x objective and an XF2046 filter set. TargetActivation v3 application was used to analyze the images and determine the number of cells in the DAPI channel, and proportion of transduced cells in the GFP channel. Data was exported to Microsoft Office Excel for statistical analysis.

### HEK293T Live Cell Assay

Cells were seeded at 40,000 cells per well. The next day, transfection with lentiviral expression plasmids with or without the addition of viral packaging plasmids was performed as described above. Plates were incubated for 21 h, medium was aspirated and replaced with PBS, containing 1% w/v BSA, 1 µg/ml Hoechst33342 and 4 ng/ml propidium iodide. Images were collected on ArrayScan in acquisition only mode to speed live-cell imaging. Images were than analyzed by rescanning with TargetActivation v3 application as above.

## Supporting Information

Figure S1 **Lentiviral human ORF overexpression library construction pipeline. Each step represents a separate series of 96-well plates.**
(TIF)

Table S1 **List of library clones.**
(XLSX)

Table S2 **Results from sequence validation of sample clones.**
(XLSX)

Table S3 **Results from transduction rate tests for very high and very low viral titer producing genes.**
(XLSX)
