# Supplementary figures and images for: Generation of a Genome Scale Lentiviral Vector Library for EF1α Promoter-Driven Expression of Human ORFs and Identification of Human Genes Affecting Viral Titer
Source: PLoS One. 2012 Dec 12;7(12):e51733. doi: 10.1371/journal.pone.0051733 (PMC3520899; doi:10.1371/journal.pone.0051733)

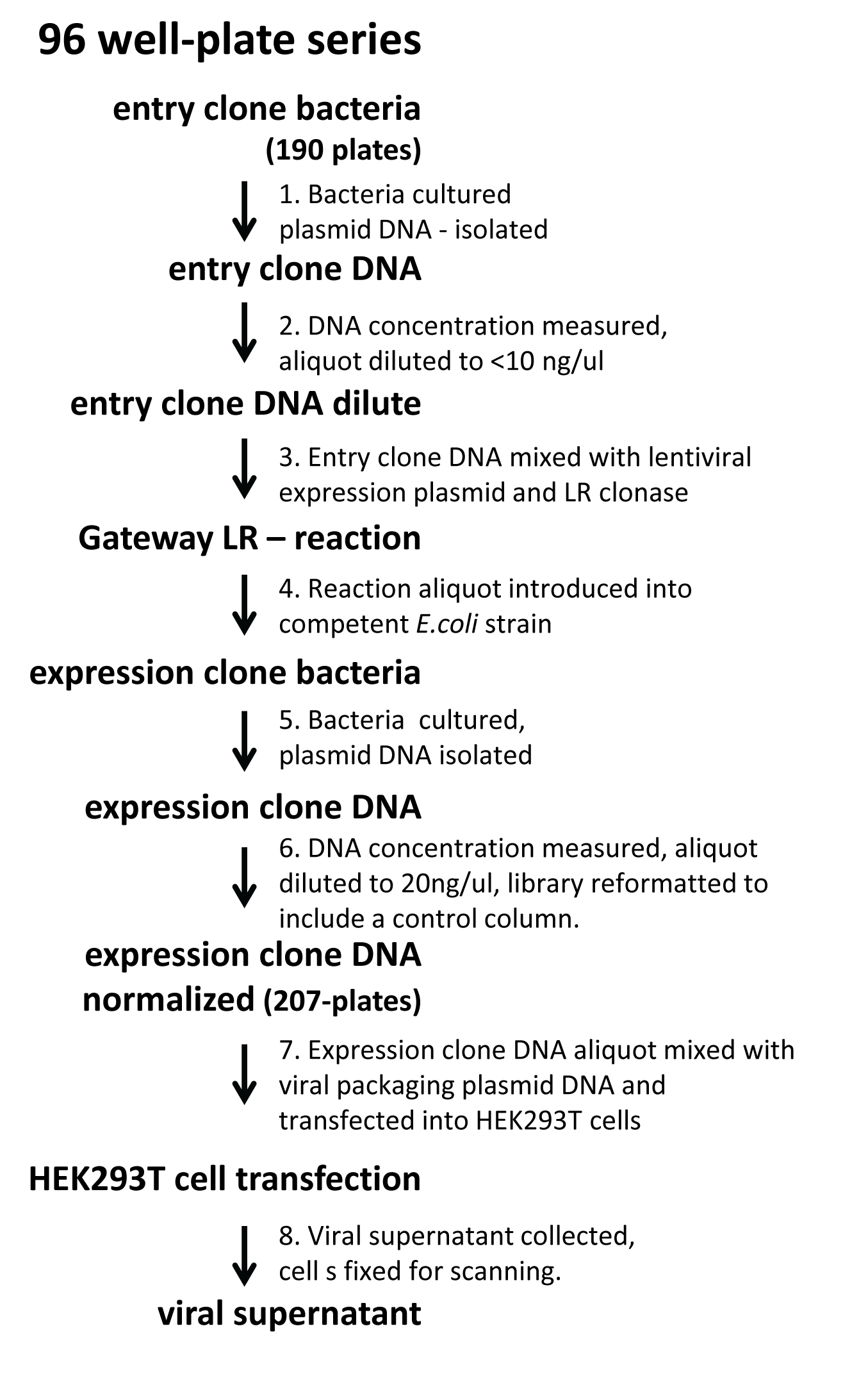

Supplement: Figure S1 — Lentiviral human ORF overexpression library construction pipeline. Each step represents a separate series of 96-well plates. (TIF) [file pone.0051733.s001.tif]
